# Screening, isolation and characterization of endophytic bacteria from upland Rice for antagonism against *Fusarium graminearum*

**DOI:** 10.3389/fmicb.2025.1717984

**Published:** 2025-12-17

**Authors:** Ying-ping Hu, Guo-dong Lu, Dong-mei Lin, Xing-sheng Lin, Hai-ling Luo, Mediatrice Hatungimana, Bin Liu, Zhan-xi Lin

**Affiliations:** 1 National Engineering Research Center of JUNCAO Technology of Fujian Agriculture and Forestry University, Fuzhou, Fujian, China; 2 National Engineering Research Center of JUNCAO Technology, Fuzhou, China; 3 Rwanda Agriculture and Animal Resources Developement Board, Kigali, Rwanda

**Keywords:** endophytes, antifungal activity, lipopeptides, volatile organic compounds, antagonisticmechanism

## Abstract

Endophytic bacteria play an important role in inhibiting plant pathogens. This study aimed to screen endophytic bacteria from upland rice with antagonistic activity against *Fusarium graminearum*, evaluated their antagonistic potential against *F. graminearum*, assessed their anti-fungal substances, and elucidated the underlying mechanisms. Some methods were performed, including dual-culture antagonism assays, lipopeptide extraction, identification of antifungal compounds via LC-MS and HS-GC-MS, metabolomic analysis, and microscopic observation. Two endophytic bacterial strains, URR1 and URR2, were identified as Pseudomonas sp. and Bacillus subtilis, respectively. Dual-culture antagonism assays demonstrated that both strains exhibited strong inhibitory activity against *F. graminearum*, with inhibition rates of 69.73% and 76.33%, respectively. In vitro experiments further revealed that bacterial suspensions at approximately 3.3 × 10 8 CFU·mL^-1^ significantly alleviated stress in upland rice seedlings infected with *F. graminearum* after 7 days. Both crude lipopeptides and volatile organic compounds (VOCs) markedly suppressed the hyphal growth of the pathogen. The maximum inhibition rate of crude lipopeptides reached 63.86% after 96 hours of treatment, while VOCs showed a peak hyphal inhibition rate of 30.38% after 48 hours of exposure. Antimicrobial lipopeptides, comprising 10 distinct surfactin isoforms and 7 fengycin variants, as well as VOCs such as acetone, ethanol, trichloromethane, pyruvic acid, and propadiene, were identified. After antagonism with lipopeptides extracted from endophytic bacteria URR2, the fungal hyphae of *F. graminearum* exhibited morphological abnormalities. Notably, treatment with URR2 resulted in the upregulation of metabolites and activation of key metabolic pathways. Metabolomic analysis indicated that the differentially upregulated metabolites encompassed a wide range of classes, including organic acids and derivatives, lipids and lipid-like molecules, organoheterocyclic compounds, organic oxygen compounds, benzenoids, organic nitrogen compounds, nucleosides, nucleotides and analogues, phenylpropanoids and polyketides, as well as lignans and neolignans. The analysis revealed that the associated molecules were significantly concentrated in multiple metabolic pathways, primarily involving ABC transporters, protein digestion and absorption, amino acid biosynthesis, aminoacyl-tRNA biosynthesis, the phosphotransferase system (PTS), starch and sucrose metabolism, among others. These results conclusively demonstrate the strong antifungal activity of strain URR2 against *F. graminearum*. The antagonistic mechanism of B. subtilis against *F. graminearum* appears to be multifaceted. Overall, our findings indicate that URR2 has promising potential to be developed as a novel biocontrol agent for the development of sustainable agriculture.

## Introduction

Phytopathogens are a major threat to the sustainability of agriculture system. Numerous fungal phytopathogens pose a significant challenge to conventional control methods due to their broad host range and soilborne nature (Summerell et al., 2003). *Fusarium graminearum* is a pathogen with a high frequnecy of wheat colonization and can cause *Fusarium* head blight (FHB) disease in wheat, causing serious harm to crop production worldwide (Khanal et al., 2021). *F. graminearum* exhibits strong pathogenicity, which relies on sophisticated infection mechanisms and remarkable environmental adaptability (Shang et al., 2024). During host invasion, the fungus secretes various extracellular enzymes to disrupt plant cell integrity (Voigt et al., 2005). As the infection progresses, it produces highly toxic secondary metabolites, such as deoxynivalenol, that not only aggravate crop disease but also compromise grain quality (Luo et al., 2025). The pathogen overwinters mainly in the form of mycelia and spores within crop residues and soil in temperate and humid regions. Surviving populations on infected debris produce ascospores, which are then dispersed by wind and rain to initiate primary infection (Leplat et al., 2016; Li et al., 2025).

Chemical fungicides are widely used in agriculture to control plant diseases. However, increasing use of chemical fungicides with high toxicity had a considerable negative impact on the sustainability of agricultural production, posing risks to the environment and human health. Biocontrol as a environment-friendly control, using antagonistic microorganisms instead of chemicals, demonstrated strong antifungal effects and harmlessness to the environment, are a practical approach to managing plant disease (Maral-Gül and Eltem, 2025). Consequently, researching and developing multifunctional biological agents—specifically those with dual functions of growth promotion and antagonism—has emerged as a pivotal area of research in sustainable agriculture. Microbial endophytes are at the forefront of sustainable agricultural practices, producing a range of bioactive compounds helping plant host respond to biotic and abiotic stress (Muhammad et al., 2024; Munakata et al., 2022; Penha et al., 2020).

Within this landscape, *Bacillus* and *Pseudomonas* are notably prominent beneficial bacteria, renowned for their plant growth-promoting properties (Baysal et al., 2024; Cueva-Yesquén et al., 2024). Both genera are capable of producing a diverse array of secondary metabolites that exhibit potent antimicrobial activities (Wang et al., 2024). Species of the genus *Bacillus* are widely marketed and utilized in modern agricultural systems. As microbial biocontrol agents, they contribute to plant health by promoting growth and suppressing various phytopathogens (Fatima et al., 2023). This approach represents a promising strategy for sustainable agriculture, as it helps eliminate the adverse environmental and human health impacts associated with chemical pesticides and fertilizers (Fessia et al., 2022; De Nunes et al., 2023; Liu et al., 2023). Generally, the genus *Bacillus* employ synergistic strategies such as biofilm formation, induced systemic resistance (ISR), and metabolic production to enhance plant resilience (Mahapatra et al., 2022). The suppressive activity of Bacillus species is primarily attributed to their production of a broad spectrum of antimicrobial agents, including hydrolytic enzymes, antibiotics, lipopeptides (LPs), and volatile metabolites (Farzand et al., 2020). *Bacillus velezensis* JCK-7158, isolated from rice, produces iturin A, surfactin, and volatile compounds as key antifungal agents. This strain serves as an eco-friendly alternative to chemical fungicides for the control of *Fusarium* head blight (Yeo et al., 2024).

As a preeminent producer of antifungal lipopeptides (e.g., surfactin, iturin, fengycin), *Bacillus* spp. exhibit potent antagonistic activity against phytopathogens such as *F. graminearum* that poses a significant threat to crops (Zalila-Kolsi et al., 2016; Palazzini et al., 2016; Iqbal et al., 2023). Among the lipopeptides, iturin and fengycin have demonstrated significant antifungal activity (Romero et al., 2007), while surfactin exhibits no marked fungitoxicity (Ongena and Jacques, 2008). Fengycin may induce pathogen cell death through mechanisms associated with membrane interaction and alterations in cell permeability (Zhang and Sun, 2018). Volatile organic compounds (VOCs) have been well investigated for their biocontrol applications (Grahovac et al., 2023). Endophytic bacteria are known to produce VOCs that can inhibit phytopathogenic fungi (Khare et al., 2018). The VOCs produced by an endophytic strain identified as *Bacillus subtilis* KRS015 exhibited significant antagonistic activity against a range of pathogenic fungi (Song et al., 2024). For instance, *Bacillus subtilis* strain DZSY21, isolated from *Eucommia ulmoides* leaves, has been shown to suppress *Curvularia lunata* through the emission of VOCs such as isopentyl acetate and 2-heptanone (Xie et al., 2020). Similarly, VOCs produced by *B. velezensis* ZSY-1 exhibited strong antifungal activity against *F. oxysporum* (Gao et al., 2017). The VOCs emitted by *Bacillus thuringiensis* G-5 exhibited significant inhibitory effects against two major postharvest pathogens of *Codonopsis pilosula*: *F. oxysporum* F-3 and *Penicillium oxalicum* F-5. These VOCs were found to markedly alter the morphology and ultrastructure of mycelia and spores, as well as compromise the integrity of the cell membranes (Mo et al., 2025).

*Bacillus* species exhibit a wide range of beneficial traits. Notably, the application of bio-fertilizers formulated with *Bacillus* bioactivators has been demonstrated to enhance the growth of upland rice (Hapsoh et al., 2021). Here, the present study aimed to isolate and evaluate endophytic bacteria from upland rice for their biocontrol potential against *F. graminearum*. Two bacterial endophytes were isolated from the roots of upland rice. Their antagonistic effects were rigorously assessed through a series of *in vitro* and *in vivo* experiments, including dual-culture antagonism assays, analyses of antifungal metabolites, evaluation of their protective efficacy in rice plants. The successful identification of effective antagonistic strains from this study could offer a sustainable and eco-friendly strategy for managing *Fusarium* head blight.

## Materials and methods

### Preparation of samples from upland rice and isolation of antagonistic bacteria

Samples were collected from upland rice field in flowering stage located in Biqiao Village, Linfang Township, Liancheng County, Longyan City, Fujian Province (longitude and latitude:116°43′52″E, 25°41′23”N). A total of 10 clusters of upland rice plants were sampled. The samples were placed in an insulated box containing ice cubes and quickly transported to the laboratory. Only roots were rinsed clean with tap water, pooled together, cut into small segments with sterile scissors, placed in sterile culture dishes, soaked in 70% alcohol for 30 s, and disinfected with a 5% (v/v) aqueous solution of sodium hypochlorite for 5 min. Finally, the samples were rinsed three times with sterile water, and the final rinsed sterile water was inoculated onto the culture medium LB plate and incubated at 30 °C for 3–5 days to check whether the sterilization was complete (Younas et al., 2023). The absence of colonies indicates that the samples were thoroughly disinfected. In a sterile mortar, 1 g of the sample were ground together with an appropriate amount of sterile quartz sand and sterile water. After grinding, the samples were transferred to a sterile test tube and made to 10 mL. The sample was shaken at 120 rpm for 30 min to obtain a sample suspension, which was sequentially diluted to prepare sample suspensions with different dilutions of 10^−1^ to 10^−6^. 100 μL of each dilution sample from 10^−3^ to 10^−6^ was taken onto the combined nitrogen fixation solid culture medium, 3 repeats for each sample. The plates were cultured upside down in incubator at 30 °C for 24–72 h. A plate with a suitable number of colonies was used to select a single colony. The strain was purified on NA (Nutrient Agar) solid culture medium using parallel streaking method with three times continuously to confirm the purified strain.

### Microorganism and culture medium

To investigate the antifungal effect, *Fusarium graminearum* PH-1 was obtained from the laboratory of the Ecological Application Research Institute of Fujian Agriculture and Forestry University. Some mediums such as LB (Luria-Bertani) medium, PDA (Potato Dextrose Agar) medium, Combined nitrogen-fixing culture medium, NA medium, Solid soybean culture medium, and MS (Murashige and Skoog) medium we used in this study. The soybean solid medium used in this experiment was adapted from the established soybean meal formula for *Bacillus subtilis* solid-state fermentation (Dai et al., 2017), with some modifications.

Detailed media formulations can be seen Supplementary Table 1. And the medium was sterilized by autoclaving at 121 °C for 20 min.

### DNA extraction, amplification of 16S rRNA and Lipopeptide biosynthesis genes

After purification on NA medium, the bacterial strain was transferred to an LB liquid medium and incubated at 30 °C with shaking at 180 r/min for 48 h. Bacterial cells were then harvested, and genomic DNA was extracted following the protocol of B518255-0100 Ezup Column Bacterial Genomic DNA Extraction Kit [Sangon Biotech (Shanghai) Co., Ltd.]. The concentration and purity of the extracted DNA were measured using a NanoDrop2000 spectrophotometer. Only DNA samples with an OD₂₆₀/OD_280_ ratio between 1.8 and 2.0 were used for polymerase chain reaction (PCR) amplification of 16S rDNA gene by specific primer of forward 16SrRNA-8F and reverse 16SrRNA-806R (Smagulova, 2024). PCR was carried out in a reaction mixture comprising bacterial DNA, 10 mM Tris–HCl, 2.5 mM MgCl₂, 50 mM KCl, 1 μM of each primer, 100 μM of each dNTP, and 1.25 U of Taq DNA polymerase. The thermal cycling protocol comprised an initial denaturation at 94 °C for 2 min, followed by 28 cycles of 94 °C for 45 s, 50 °C for 45 s, and 72 °C for 90 s. The lipopeptide biosynthesis genes sfp and FenD were amplified by polymerase chain reaction (PCR) using gene-specific primers. The sfp gene was amplified with the primer pair Sfp-f (5′-ATGAAGATTTACGGAAATTTA-3′) and Sfp-r (5′-TTATAAAAGCTCTTCGTACG-3′) (Hsieh et al., 2004), while the FenD gene was amplified using primers FNDF2 (CTGGGAGGTCAGCCGGTCTG) and FNDR2 (GTGGTCGCCGGTTCACAAAT) (Cao et al., 2012).

The amplified products were quantified using the Qubit dsDNA HS Assay Kit and subjected to Illumina sequencing. The resulting sequences were analyzed for homology using the BLAST program from the National Center for Biotechnology Information (NCBI).^1^ Endophytic species were identified based on the highest query coverage and BLAST scores (Sampangi-Ramaiah et al., 2020). Phylogenetic analysis was performed using MEGA 11 software, with parameter settings including maximum likelihood statistical method, bootstrap method of phylogeny test, 1,000 for bootstrap replications.

### Extraction and component determination of crude lipopeptide from antagonist

#### Crude extract of lipopeptide

A single purified colony of isolated strain was inoculated into sterile LB liquid medium and incubated at 30 °C with shaking at 200 r/min for 12 h to prepare the seed culture. Subsequently, 1% (v/v) of the seed culture was transferred into fresh LB medium and cultivated under the same conditions (30 °C, 200 r/min) for 48 h. The fermentation broth was centrifuged at 12,000 rpm for 20 min at 4 °C to collect the supernatant. The methods of lipopeptides precipitation and collection are as previously described (Chen et al., 2008), with the following modifications: the supernatant was then acidified to pH 2.0 using 6 mol/L HCl and stored overnight at 4 °C. The resulting mixture was centrifuged at 8,000 r/min for 20 min to collect the precipitate. The precipitate was resuspended in an equal volume of 10% methanol, and the pH was adjusted to 7.0 with 1.0 M NaOH. After further centrifugation at 8,000 r/min for 20 min, the supernatant was collected and filtered through a 0.22 μm microporous membrane to obtain the crude lipopeptide extract.

#### Determination of crude lipopeptide

A 500 μL aliquot of the crude antimicrobial peptide extract was centrifuged at 13,000 r/min for 10 min, and the resulting supernatant was collected for LC–MS (Liquid Chromatography-Mass Spectrometry) analysis. Separation was performed on an UltiMate 3,000 UHPLC system equipped with a C18 column (1.9 μm, 2.1 mm × 100 mm). The mobile phase consisted of 0.1% formic acid in water (A) and 0.1% formic acid in acetonitrile (B), with a flow rate of 0.3 mL·min^−1^ and an injection volume of 10 μL. The gradient elution program used is detailed in Table 1.

**Table 1:** Mobile phase gradient elution procedure.

Time (min)	A(0.1% FA/water)	B (0.1% FA/ACN)
0	95	10
15	0	100
17	0	100
17.1	95	10
20	95	10

Mass spectrometric analysis was performed using a Q-Exactive instrument (Thermo Fisher Scientific, CA, United States) equipped with a HESI ion source. The source temperature was set to 310 °C and the capillary temperature to 320 °C. Sheath gas and auxiliary gas flow rates were maintained at 30 and 10 arbitrary units, respectively. The spray voltage was set to 3.0 kV in positive ion mode and 2.8 kV in negative ion mode. Data were acquired in data-dependent acquisition (DDA) mode with a loop count of 10. HCD fragmentation was performed using stepped normalized collision energies of 10, 28, and 35 eV. Full MS scans were acquired over the m/z range of 80–1,200 with a resolution of 70,000, an AGC target of 3 × 10^6^, and a maximum injection time of 200 ms. MS/MS scans were performed with a resolution of 17,500, an AGC target of 1 × 10^5^, and a maximum injection time of 50 ms. The final identification of the lipopeptide compounds was achieved by comparing the detected peak characteristics with those reported in existing literature (Jemil et al., 2017; Govaerts et al., 2002).

### Detection of volatile compounds in endophytic bacteria by headspace gas chromatography–mass spectrometry

Volatile compounds were collected using tedlar bags and analyzed by a pre-concentration system (Entech 7,200, Entech, United States) coupled with gas chromatography–mass spectrometry (7890B-5977A, Agilent Technologies, United States). Specifically, the tedlar bag was connected to the pre-concentration unit, from which a 300 mL sample was drawn. The sample was then concentrated and focused through a three-stage cold trap to remove moisture, nitrogen, and carbon dioxide, before being introduced into the gas chromatograph (GC). Separation of target compounds was achieved using a capillary column with temperature programming, and detection was carried out by mass spectrometry (MS).

The pre-concentration parameters were set as follows: the sample first entered the primary cold trap (−40 °C) for initial trapping of target compounds. The primary trap was then rapidly heated to 10 °C, and the analytes were transferred via helium carrier gas to the secondary cold trap (−40 °C). Subsequently, the secondary trap was flash-heated to 180 °C, and the sample was moved to the tertiary cold trap (−160 °C). Finally, the tertiary trap was rapidly heated to 80 °C to inject the target compounds into the GC. Key GC–MS parameters were detailed in Table 2.

**Table 2:** Key GC–MS parameters.

Parameter type	Setting
Chromatographic column	HP-1 (60 m × 250 μm × 0.32 μm)
Carrier gas	Helium
Flow rate	4 mL·min^−1^ (constant)
Oven temperature program	
- Initial	10 °C, held for 3 min
- Ramp 1	5°C·min^−1^ to 120 °C
- Ramp 2	10 °C·min^−1^ to 250 °C, held for 7 min
Ionization mode	Electron Impact (EI)
Ionization energy	70 eV
Ion Source temperature	230 °C
Quadrupole temperature	150 °C
Acquisition mode	Full Scan (Scan)

### Antagonistic experiment

#### Dual-culture antagonism

*Fusarium graminearum* was cultured on PDA solid medium and incubated at 28 °C for 48 h in a constant temperature incubator. Samples were taken when the mycelium had nearly covered the agar plate. Each tested bacterial strain was activated in LB liquid medium and incubated in a shaker at 30 °C and 180 r/min for 24 h before sampling. Using a pipette, 10 microliters of the bacterial suspension were spotted onto LB solid medium. A 5 mm diameter agar plug of *F. graminearum* was then inoculated 2 centimeters away from the bacterial spot to initiate the confrontation assay. All plates were incubated at 28 °C for 72 h. The antagonistic activity of each bacterial strain was observed and recorded. The inhibition ratios of mycelium growth of *F. graminearum* D187 were calculated with the following formula (Zhou et al., 2011). Inhibition ratio (%) = (C - T)/C × 100%, where C is the diameter of the control colony and T is the diameter of the treatment colonies.

### Determination of the antagonistic effects of crude lipopeptide extracts on *Fusarium graminearum*

#### Antagonism test

A 100 mL aliquot of PDA medium was prepared and sterilized. Once the medium had cooled to 50–60 °C, 1 mL of sterile crude lipopeptide extract was added and mixed thoroughly to prepare a PDA plate containing 1% (v/v) crude lipopeptide extract. After solidification, a 5 mm diameter agar plug of *F. graminearum* was inoculated at the center of the plate. A control plate was prepared similarly using PDA medium without the lipopeptide extract and inoculated with an identically sized fungal plug. Each treatment was triplicate. All plates were incubated at 28 °C, and hyphal growth of *F. graminearum* was recorded at 24 h, 48 h, 72 h, and 96 h.

#### Microscopic observation of *Fusarium graminearum* hyphae

A fungal plug of *F. graminearum* was inoculated at the center of a PDA plate. Then, 20 μL of crude lipopeptide extract was applied to multiple points 2 cm away from the fungal plug. As a control, 20 μL of sterile LB solution was applied in the same manner at separate locations. The plate was sealed and incubated at 28 °C for 2 days. After incubation, the mycelium of pathogenic fungi were collectedd, and the hyphae were moistened with sterile water on a glass slide. The morphology of the hyphae was then examined under a microscope.

#### Determination of the antagonistic effects of volatile compounds on *Fusarium graminearum*

Two PDA plates were prepared for co-cultivation. On one plate, 200 μL of the bacterial suspension was inoculated and evenly spread. On the other plate, a 5 mm diameter agar plug of *F. graminearum* was placed with the mycelial side facing downward. The two plates were then sealed together and incubated at 28 °C for 48 h. A control group was set up using 200 μL of germ-free LB liquid instead of the bacterial suspension. Each treatment was replicated three times. The hyphal growth diameter was measured and recorded at 24 h, 48 h, 72 h, and 96 h.

#### Antagonistic effects *in vitro* against *Fusarium graminearum* infection in upland rice seedlings

Strains URR1 and URR2 were pre-activated by inoculating 1 mL of each bacterial culture into 100 mL of fresh sterile LB liquid medium, followed by incubation in a shaker at 30 °C and 180 rpm for 24 h. The bacterial cultures were then mixed in a 1:1 ratio (3 mL URR1 + 3 mL URR2). From this mixture, 3 mL was taken and diluted with sterile water to prepare a 3% (v/v) mixed bacterial suspension (approximately 3.3 × 10^8^ cfu·mL^−1^), which served as the treatment group. A control group was established using sterile water.

For upland rice seed germination, seeds were soaked in water for 2 days, surface-sterilized with sodium hypochlorite and 75% ethanol, and placed in sterile glass culture dishes. The treatment group received 20 mL of the mixed bacterial suspension, while the control group received an equal volume of sterile water. All dishes were kept in a laminar flow hood under a 12-h light/dark cycle at 25 °C. Each treatment was triplicate. After 17 days of germination, 30 seedlings per treatment were selected for the infection assay. The root length of each seedling was measured prior to infection. Seedlings were then transferred to sterile culture dishes, with 10 seedlings per dish. Each dish received 15 mL of sterilized MS (Murashige and Skoog) liquid medium, one 5 mm agar block of *F. graminearum*, and 3 mL of *F. graminearum* spore suspension. The preparation of fungal spore suspension follows protocol: After the mycelium of *F. graminearum* had fully colonized the PDA medium, nine mycelial plugs (approximately 0.5 cm in diameter) were obtained using a cork borer and transferred into potato dextrose broth (PDB). The cultures were then incubated in a shaking incubator at 25 °C and 170 rpm for 5 days to promote the production of macroconidia. The resulting culture was filtered through a double layer of sterile gauze to remove mycelial debris, and the filtrate was centrifuged at 4 °C and 5,000 × g for 15 min to collect the spores. After discarding the supernatant, the spore pellet was resuspended in half-strength potato dextrose broth (½ PDB). The spore concentration was determined using a hemocytometer under a microscope and adjusted to a final density of 1.25 × 10^4^ spores·mL^−1^ with ½ PDB. The spore suspension was stored at 4 °C for subsequent use.

The treatment group was supplemented with 3 mL of the 3% mixed bacterial suspension, while the control received 3 mL of sterile water. All dishes were incubated in a laminar flow hood at room temperature for 7 days. An additional 15 mL of MS nutrient solution was added once during this period. After incubation, the main and lateral root lengths of each upland rice seedling were measured again.

### Metabolomic analysis of endophytic bacteria antagonizing *Fusarium graminearum*

#### Samples collection and preparation

A 100 μL aliquot of the URR2 bacterial suspension was evenly spread on an solid soybean culture medium plate and incubated at 30 °C until full growth was achieved. A sterile scalpel was used to excise a strip of bacterial lawn measuring 2 mm in width and 6 cm in length, which was then placed in the center of a soybean medium plate. Pre-grown *F. graminearum* strips of the same dimensions were placed on both sides of the bacterial strip, positioned 2 cm from the center with the mycelial side facing downward, to initiate confrontation culture. A control was prepared using blank LB medium strips under the same conditions.

**Figure 1:**
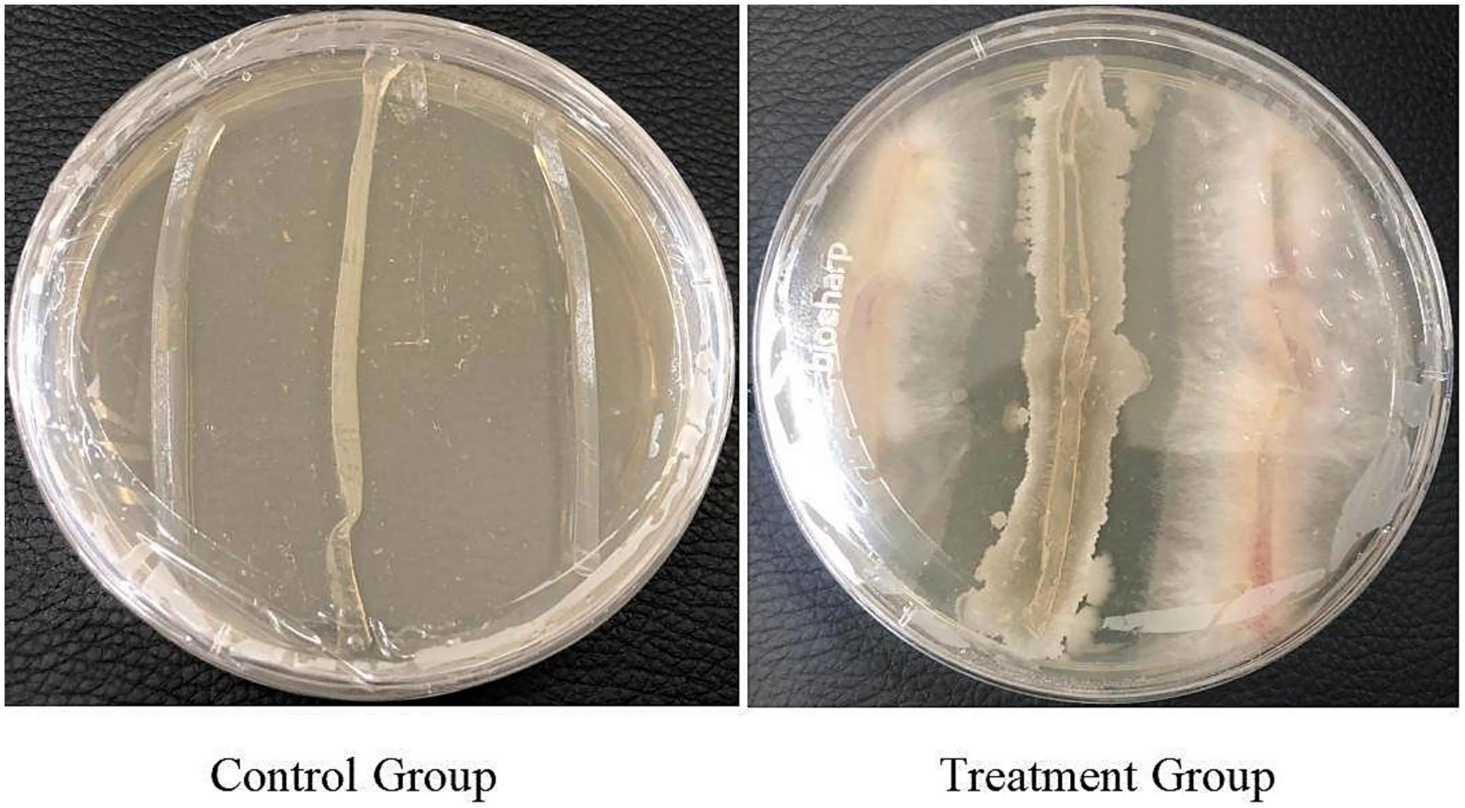
Samples’ preparation.

The plates were incubated at 28 °C for 5 days, after which samples were collected. The treatment group sample was designated as URR2 and the control as CK, respectively (refer to Figure 1). For sampling, a sterile, enzyme-free 1.5 mL centrifuge tube was pre-cooled in liquid nitrogen. Bacterial cells of URR2 were quickly scraped from the solid medium using a sterile inoculation loop and transferred into the pre-cooled tube. The tube was immediately wrapped in aluminum foil, flash-frozen in liquid nitrogen, and stored at −80 °C until further analysis.

The culture medium was aspirated from the samples using a pipette. The cells were then washed with pre-warmed PBS (37 °C), after which the PBS was removed. To precipitate proteins and extract metabolites, 800 μL of cold methanol/acetonitrile (1:1, v/v) was added. The mixture was transferred to a new centrifuge tube and centrifuged at 14,000 g for 20 min. The resulting supernatant was collected and dried in a vacuum centrifuge. For LC – MS analysis, the dried samples were reconstituted in 100 μL of acetonitrile/water (1,1, v/v), followed by centrifugation at 14,000 g and 4 °C for 15 min. The final supernatant was injected into the LC – MS system.

#### LC–MS/MS analysis

Thw analysis was performed using a UHPLC system (Agilent 1,290 Infinity LC) coupled with a quadrupole time-of-flight mass spectrometer (AB Sciex TripleTOF 6,600). Chromatographic separation was carried out on a HILIC column (ACQUITY UPLC BEH Amide, 2.1 × 100 mm, 1.7 μm) with a gradient of acetonitrile and ammonium acetate/ammonium hydroxide solution at 0.5 mL/min. Electrospray ionization was operated in both positive and negative modes. Full-scan MS data were collected from m/z 60–1,000, and auto MS/MS fragmentation was triggered by information-dependent acquisition using a collision energy of 35 ± 15 eV. Compound identification was confirmed by comparing retention times and fragmentation patterns with those of authentic standards or literature spectra.

### Data statistical analysis

Data processing was conducted using IBM SPSS Statistics 26 and Origin 2021 software. A *p*-value of ≤ 0.05 was considered statistically significant. Student’s *t* test was applied to determine the significance of differences between two groups of independent samples. Significantly altered metabolites were identified based on a variable importance in the projection (VIP) value greater than 1 and a *p*-value less than 0.05. Additionally, Pearson’s correlation analysis was conducted to assess the relationship between two variables. Experiments were conducted in triplicate for each variant.

## Results

### Isolation, identification of *Fusarium graminearum*-antagonistic endophytic bacteria and dual-culture antagonism

**Figure 2:**
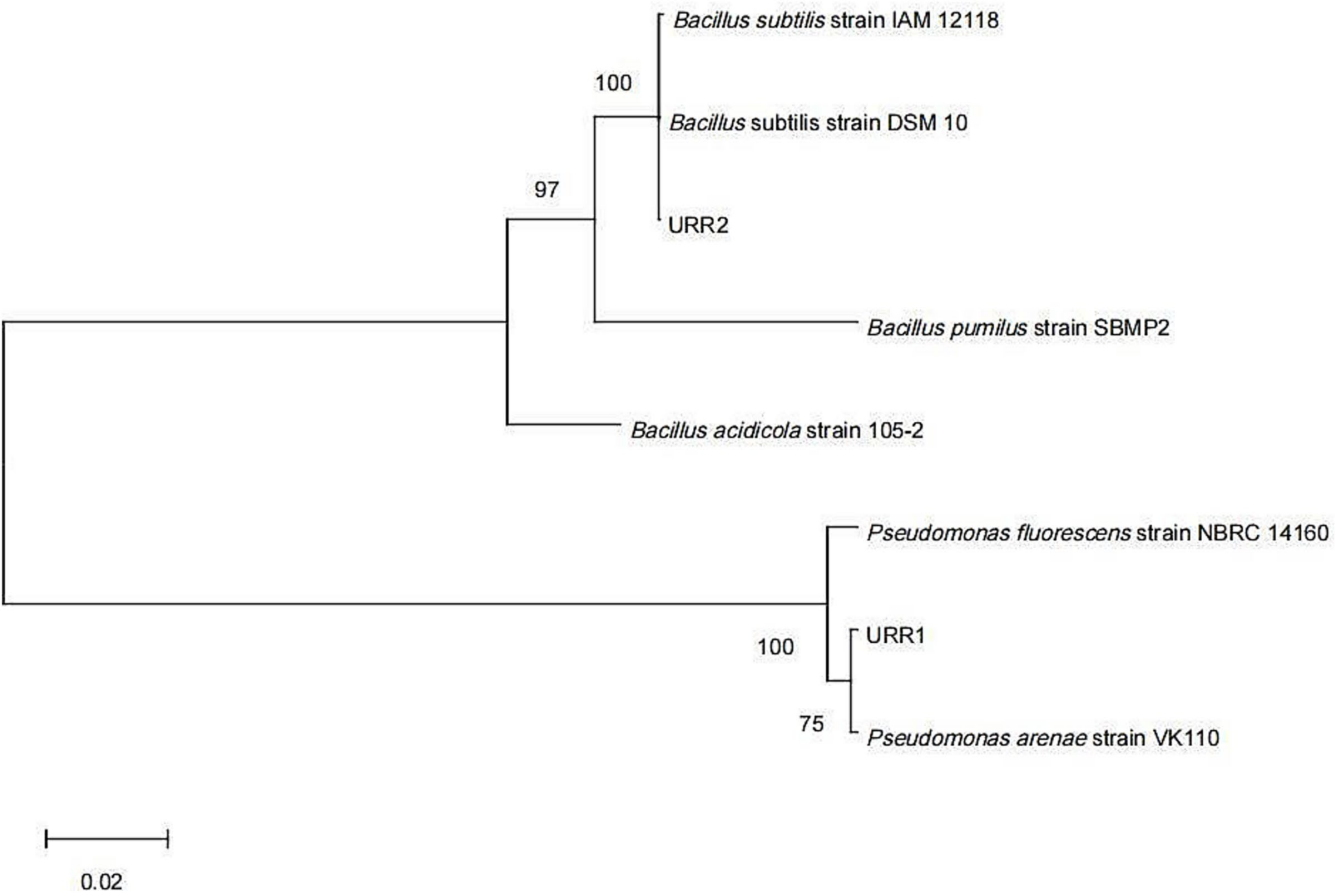
The phylogenetic tree of URR1 and URR2 was constructed with MEGA11 software using the maximum likelihood method with 1,000 bootstrap replicates.

**Figure 3:**
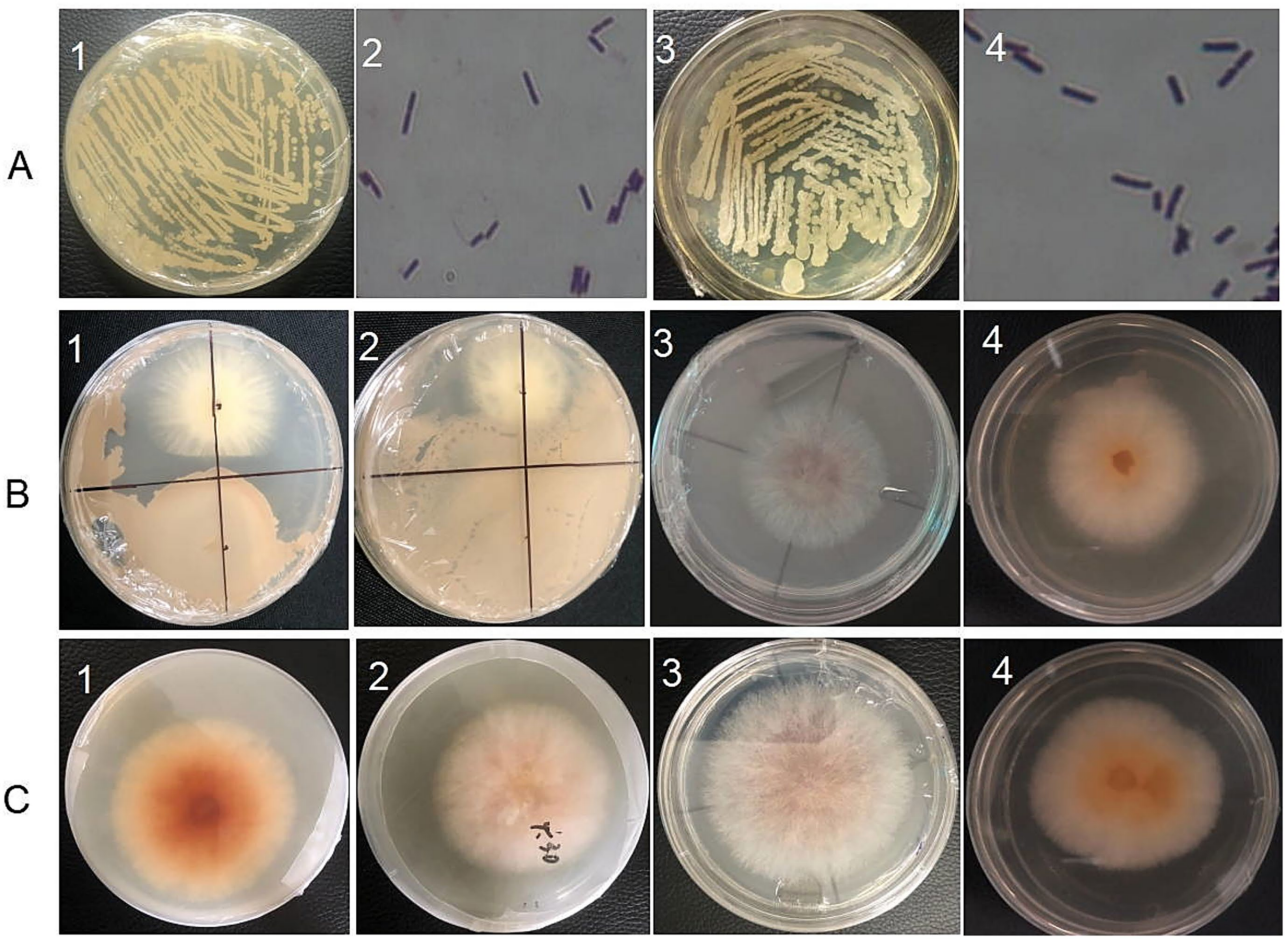
Morphology of isolated strains and their antagonistic effects on hyphal growth of *F. graminearum*. **(A1)** Colonial morphology of URR1; **(A2)** Gram staining test of URR1 (the magnification of the microscope is 100x); **(A3)** colonial morphology of URR2; **(A4)** Gram staining test of URR2 (the magnification of the microscope is 100x); **(B1)** antagonistic test of strain URR1 against *F. graminearum*; **(B2)** antagonistic test of strain URR2 against *F. graminearum*; **(B3)** antagonistic effect of URR2 crude lipopeptide on *F. graminearum*; **(B4)** antagonistic effect of URR2 VOCs on *F. graminearum*; **(C1)** hyphal growth of *F. graminearum* without antagonistic effect of URR1; **(C2)** hyphal growth of *F. graminearum* without antagonistic effect of URR2; **(C3)** hyphal growth of *F. graminearum* without crude lipopeptide of URR2; **(C4)** hyphal growth of *F. graminearum* without VOC of strain URR2.

**Figure 4:**
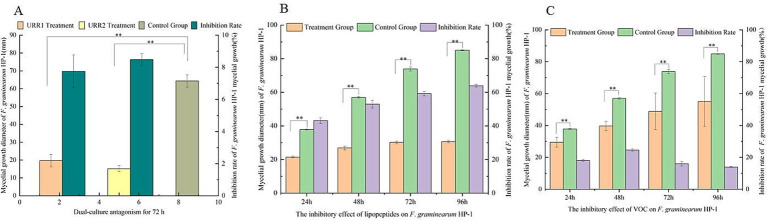
Determination of antagonistic effects of endophytic bacteria, its lipopeptide, and VOC on *F. graminearum* PH-1. **(A)** Dual-culture antagonism for 72 h among different treatment. **(B)** Antagonistic effects of crude lipopeptide extracts from URR2 on *F. graminearum*. **(C)** Antagonistic effects of VOC from URR2 on *F. graminearum.*

In this study, two endophytic bacterial strains, designated URR1 and URR2, were isolated from the root tissues of upland rice. Based on phylogenetic analysis, URR1 was identified as belonging to the genus *Pseudomonas*, while URR2 was classified as *Bacillus subtilis*. The phylogenetic tree illustrating their relationships is presented in Figure 2. Strain URR1 was identified as Gram-negative, exhibiting a purplish-red color after Gram staining, whereas Strain URR2 was Gram-positive, showing a purple color under the same staining conditions. Their colonial morphology and Gram staining results are depicted in Figure 3A. Furthermore, the two strains demonstrated significant antifungal activity (*p* < 0.01) against *F. graminearum* (Figures 3B,C, B1,C1,B2,C2). The inhibition rate reached 69.73% for URR1 and 76.33% for strain URR2 (see Figure 4A).

### Endophytic bacteria alleviate the impact of *Fusarium graminearum* infection on upland rice seedlings

**Figure 5:**
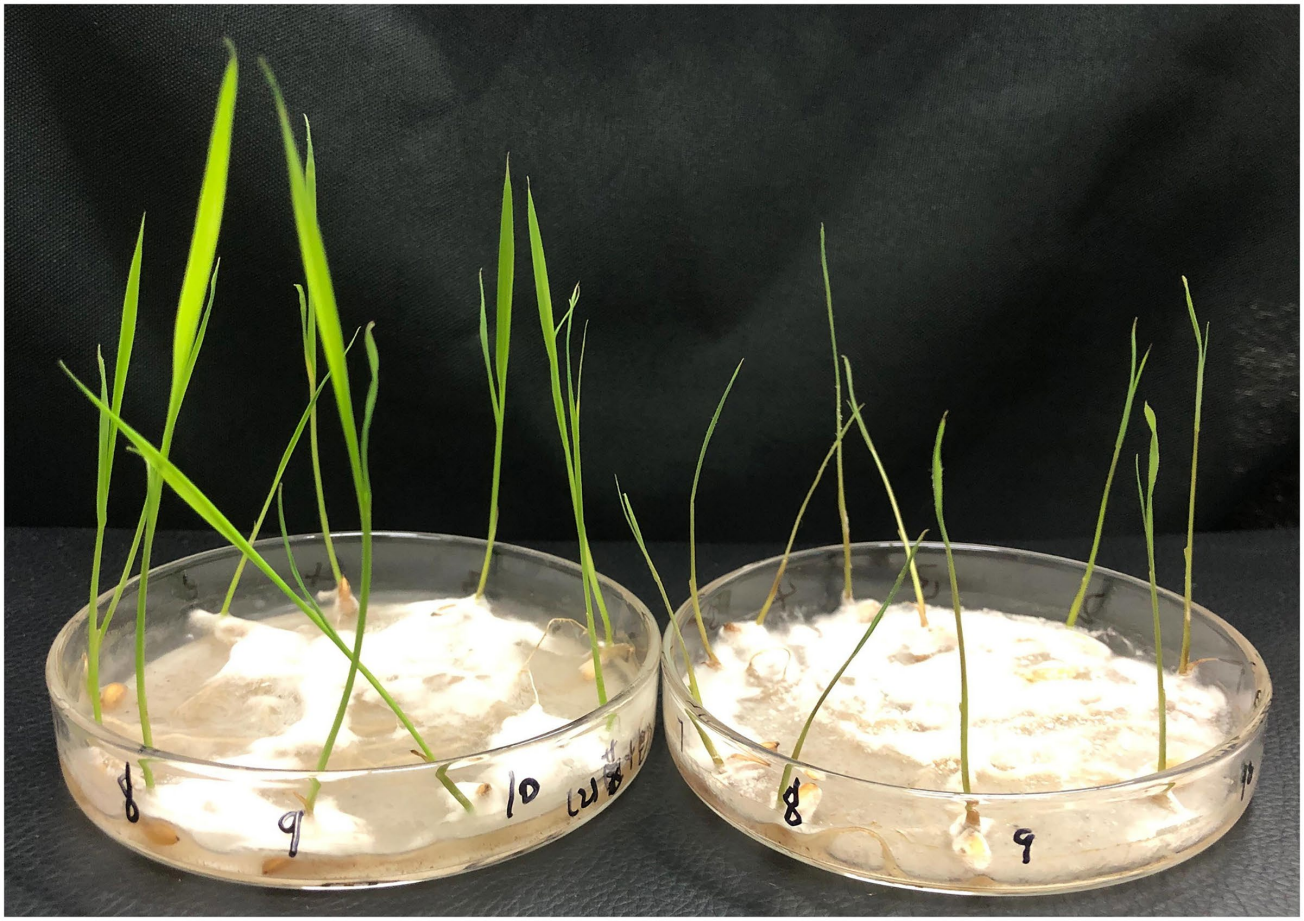
The antagonistic effect of mixed bacterial suspension (left culture dish) and control group (right culture dish) on the growth of upland rice seedlings infected with *F. graminearum* PH-1.

After 7 days of infection with *F. graminearum*, upland rice seedlings in culture dishes without antagonistic bacterial supplementation exhibited growth inhibition and leaf wilting. In contrast, the addition of a 3% mixed bacterial suspension (approximately 3.3 × 10^8^ CFU·mL^−1^) significantly antagonized the pathogen, and the plants displayed normal growth (Figure 5). Specifically, the axial root length and secondary root length of plants in the treatment group reached 90.50 mm and 48.42 mm, respectively, whereas those in the control group were only 60.60 mm and 23.93 mm. The differences in both axial and secondary root lengths between the treatment and control groups were highly significant (*p* < 0.01). Furthermore, the average increases in axial and secondary root lengths in the treatment group after infection were 10.37 mm and 9.27 mm, respectively. In the control group, the axial root showed an average increase of 5.22 mm, while the secondary root length decreased by an average of 2.36 mm. This negative change was attributed to the breakage of secondary roots caused by the pathogen infection.

## Results of metabolomic analysis

### Metabolite identification and chemical taxonomy

**Figure 6:**
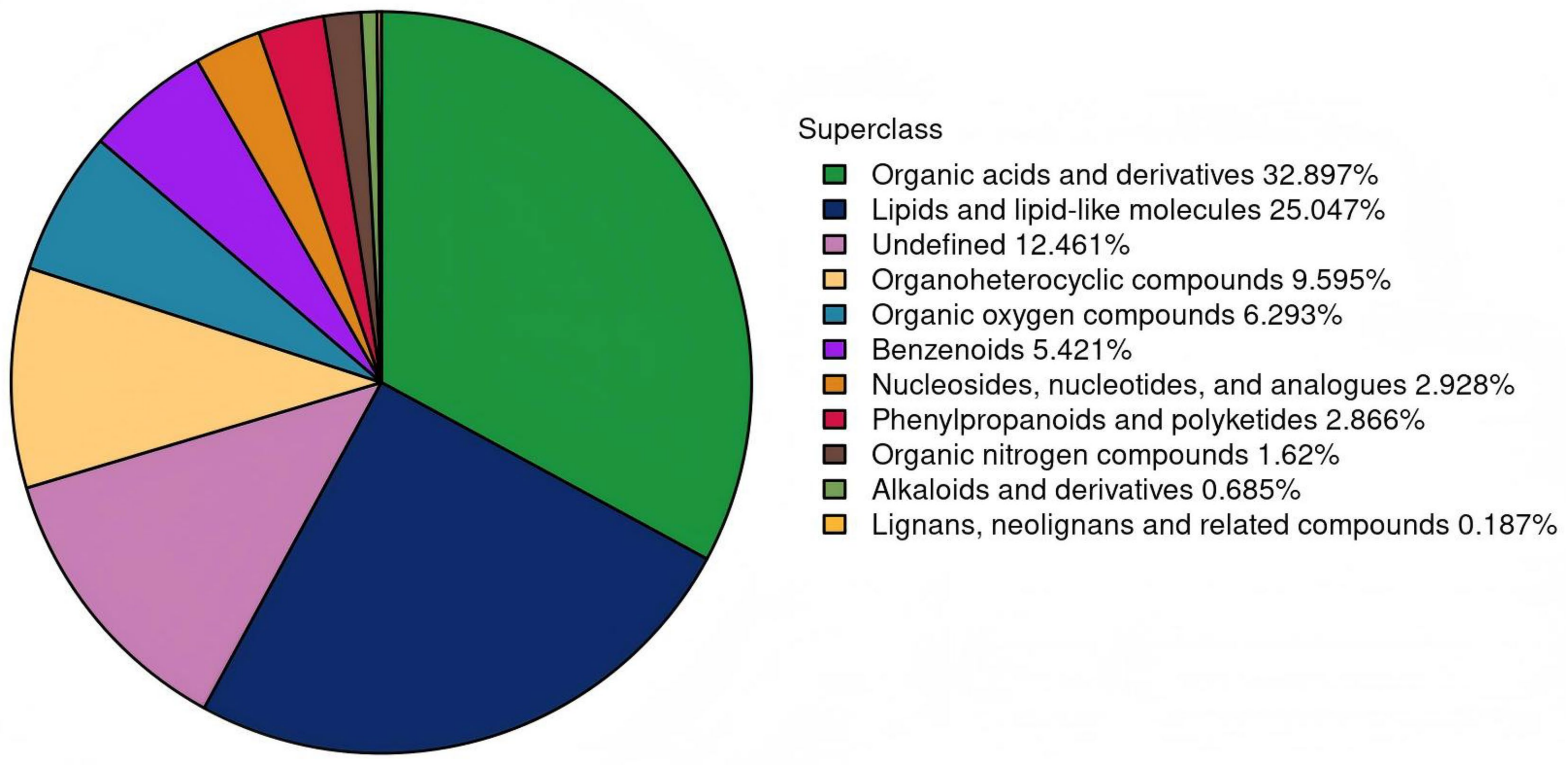
The proportion of metabolites identified in each chemical classification. The different color blocks in the figure mean different chemical classification items, and the percentage means the percentage of identified metabolites in a chemical classification item to all identified metabolites. Metabolites without chemical classification are defined as undefined.

The metabolites were identified by matching their retention times, precise molecular masses (mass error < 10 ppm), and secondary fragmentation spectra against a locally established standard database (in-house database, Shanghai Applied Protein Technology), as previously described (Luo et al., 2017; Gu et al., 2018). In the present study, a total of 1,605 metabolites were identified through integrated analysis of both positive and negative ion modes. Among these, 1,074 metabolites were detected in positive ion mode and 531 in negative ion mode. Based on chemical taxonomy, these metabolites were classified into 10 distinct superclasses: organic acids and derivatives (32.897%), lipids and lipid-like molecules (25.047%), organoheterocyclic compounds (9.595%), organic oxygen compounds (6.293%), benzenoids (5.421%), nucleosides, nucleotides and analogs (2.928%), phenylpropanoids and polyketides (2.866%), organic nitrogen compounds (1.62%), alkaloids and derivatives (0.685%), and lignans, neolignans and related compounds (0.187%). Notably, organic acids and derivatives together with lipids and lipid-like molecules represented the majority of the identified metabolites, collectively accounting for 57.95% of the total (Figure 6). Furthermore, the top 15 most abundant metabolite classes were identified as: carboxylic acids and derivatives, prenol lipids, organooxygen compounds, fatty acyls, steroids and steroid derivatives, glycerophospholipids, benzene and substituted derivatives, indoles and derivatives, peptidomimetics, organonitrogen compounds, flavonoids, imidazopyrimidines, benzopyrans, pyridines and derivatives, and pyrimidine nucleotides.

### Differential metabolite analysis

**Figure 7:**
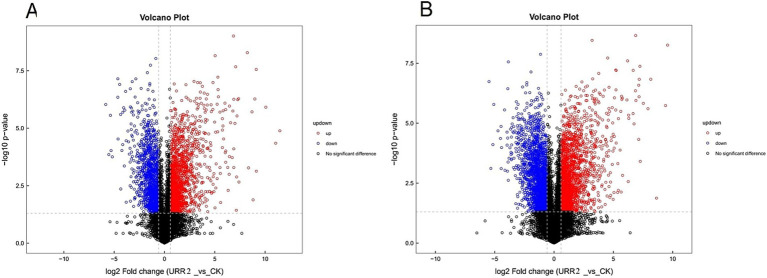
Volcano diagram of differential metabolite ion. **(A)** Volcano diagram of negative ion mode **(B)** volcano diagram of positive ion mode (red represents upregulation, blue represents downregulation, black represents no difference). The value of horizontal axis in the figure is the logarithm of log2 for fold change, and the value of vertical axis is the logarithm of -log10 when *p* value is less than 0.05. Metabolites significantly upregulated when FC value is more than 1.5 and *p* value is less than 0.05 are indicated in rose red, while metabolites significantly downregulated when FC value is less than 0.67 and *p* value is less than 0.05 are indicated in blue. Non significantly different metabolites are indicated in black.

Common univariate statistical methods for analyzing differences between two sample groups include fold change (*FC*) analysis and t-tests or non-parametric tests. Differential analysis was conducted on all detected metabolites (including unidentified compounds) in both positive and negative ion modes using the thresholds: *FC* > 1.5 for upregulation, *FC* < 0.67 for downregulation, and a *p*-value < 0.05. Metabolites satisfying these criteria were visualized in volcano plots (Figure 7). The plots showed a greater number of upregulated metabolites (represented by red points) compared to downregulated metabolites (blue points) in both ionization modes. This pattern implies that the antagonistic interaction triggered substantial metabolic upregulation in *B. subtilis* URR2, possibly as part of a stress response or adaptive mechanism.

Differential upregulation of metabolites was observed across multiple compound classes in both positive and negative ion modes, with the following distribution: Organic acids and derivatives (94 positive ions, 34 negative ions), Lipids and lipid-like molecules (54 positive ions, 52 negative ions), Organoheterocyclic compounds (22 positive ions, 19 negative ions), Organic oxygen compounds (17 positive ions, 5 negative ions), Benzenoids (10 positive ions, 4 negative ions), Organic nitrogen compounds (7 positive ions, 8 negative ions), Nucleosides, nucleotides, and analogs (7 positive ions, 7 negative ions), Phenylpropanoids and polyketides (3 positive ions, 3 negative ions), and Lignans, neolignans and related compounds (1 positive ion, 1 negative ion).

In the negative ion mode, the significantly upregulated differential metabolites are mainly arctigenin, hederacoside C, fulvestrant 9-sulfone, 1-(9Z,12Z-octadecadienoyl)-2-hydroxy.

**Figure 8:**
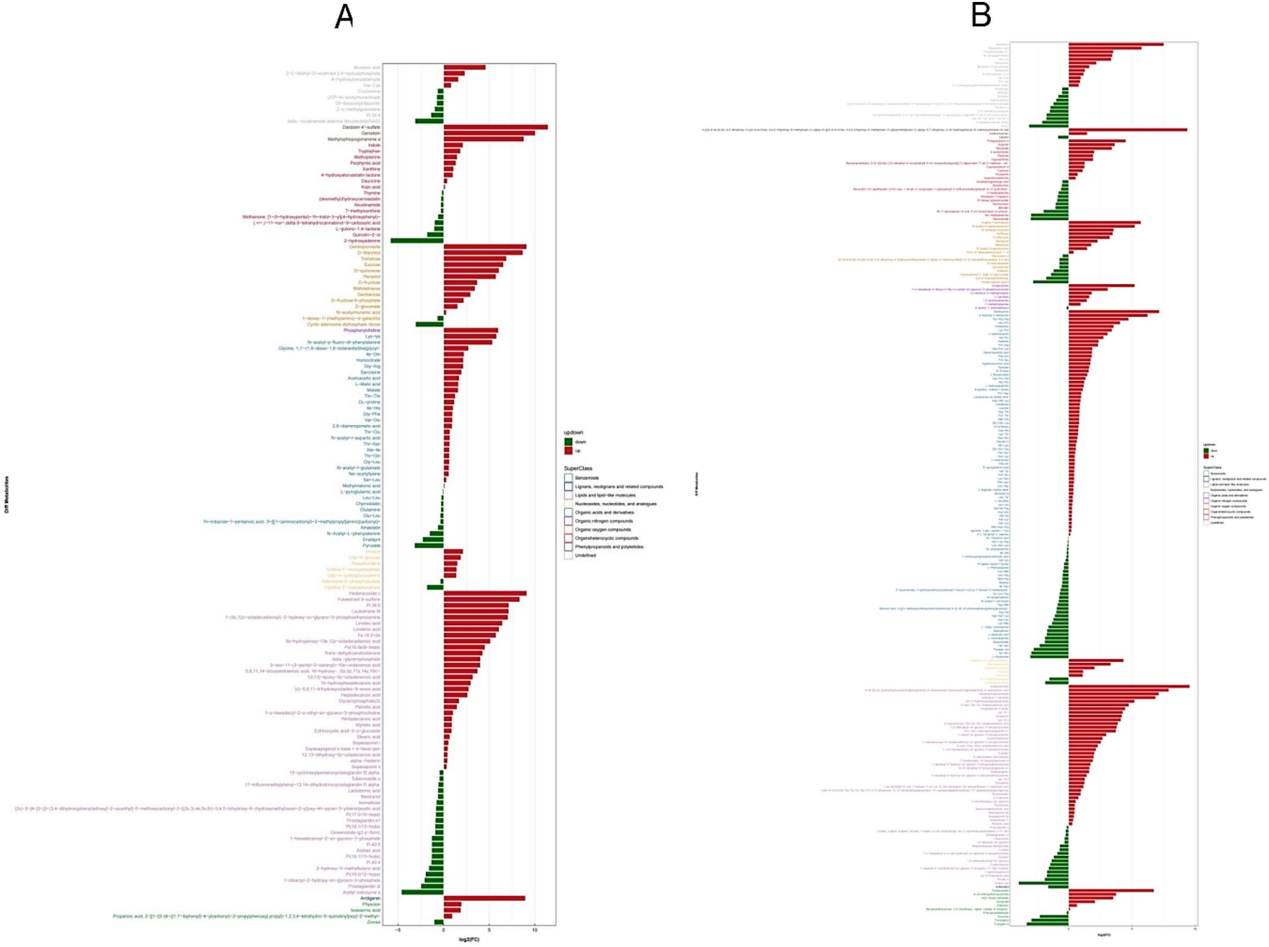
Analysis of significantly altered metabolites in **(A)** negative and **(B)** positive ion modes. The horizontal axis represents the log₂ fold-change (FC) values, indicating the magnitude of abundance changes. The vertical axis lists the significantly differential metabolites. Metabolites highlighted in red and green denote upregulation and downregulation, respectively.

-sn-glycero-3-phosphoethanolamine, leukotriene F4, linoleic acid, linolenic acid, 9S-hydroperoxy-10E,12Z-octadecadienoic acid, Lys-Lys, N-acetyl-p-fluoro-DL-phenylalanine, phosphorylcholine, gentiopicroside, D-mannitol, trehalose, sucrose, D-quinovose, perseitol, daidzein 4′-sulfate, genistein, methylophiopogonanone A, muramic acid, etc. In contrast, the significantly downregulated differential metabolites primarily comprise acetyl coenzyme A, nicotinamide adenine dinucleotide (NAD), 2-hydroxyadenine, cyclic adenosine diphosphate ribose, etc (Figure 8A).

In the positive ion mode, the significantly upregulated differential metabolites primarily consist of tropacocaine, acetylcarnitine, oxobutanoic acid, glycerophosphocholine, isobutyryl-L-carnitine, (2R)-3-hydroxyisovaleroylcarnitine, stachydrine, 4-hydroxy -L-isoleucine, acetylcholine, alpha-mannobiose, N-acetyl-D-galactosaminitol, 3-[(2S,3R,4S,5S,6R)-4,5-dihydroxy-3-[(2R,3R,4R,5R,6S)-3,4,5-trihydroxy-6-methyloxan-2-yl]oxy-6-[[(2R,3R,4R,5R,6S)-3,4,5-trihydroxy-6-methyloxan-2-yl]oxymethyl]oxan-2-yl]oxy-5,7-dihydroxy-2-(4-hydroxyphenyl)-6-methoxychromen-4-one, mexiletine, stearidonic acid, among others. Conversely, the significantly downregulated differential metabolites mainly include acetyl-CoA, NAD^+^, niacinamide, hexapropylene glycol, pyroglu-val, Tyr-Glu, L-glutamate, frangulin A, etc (Figure 8B).

### Enrichment analysis of KEGG metabolic pathway

**Figure 9:**
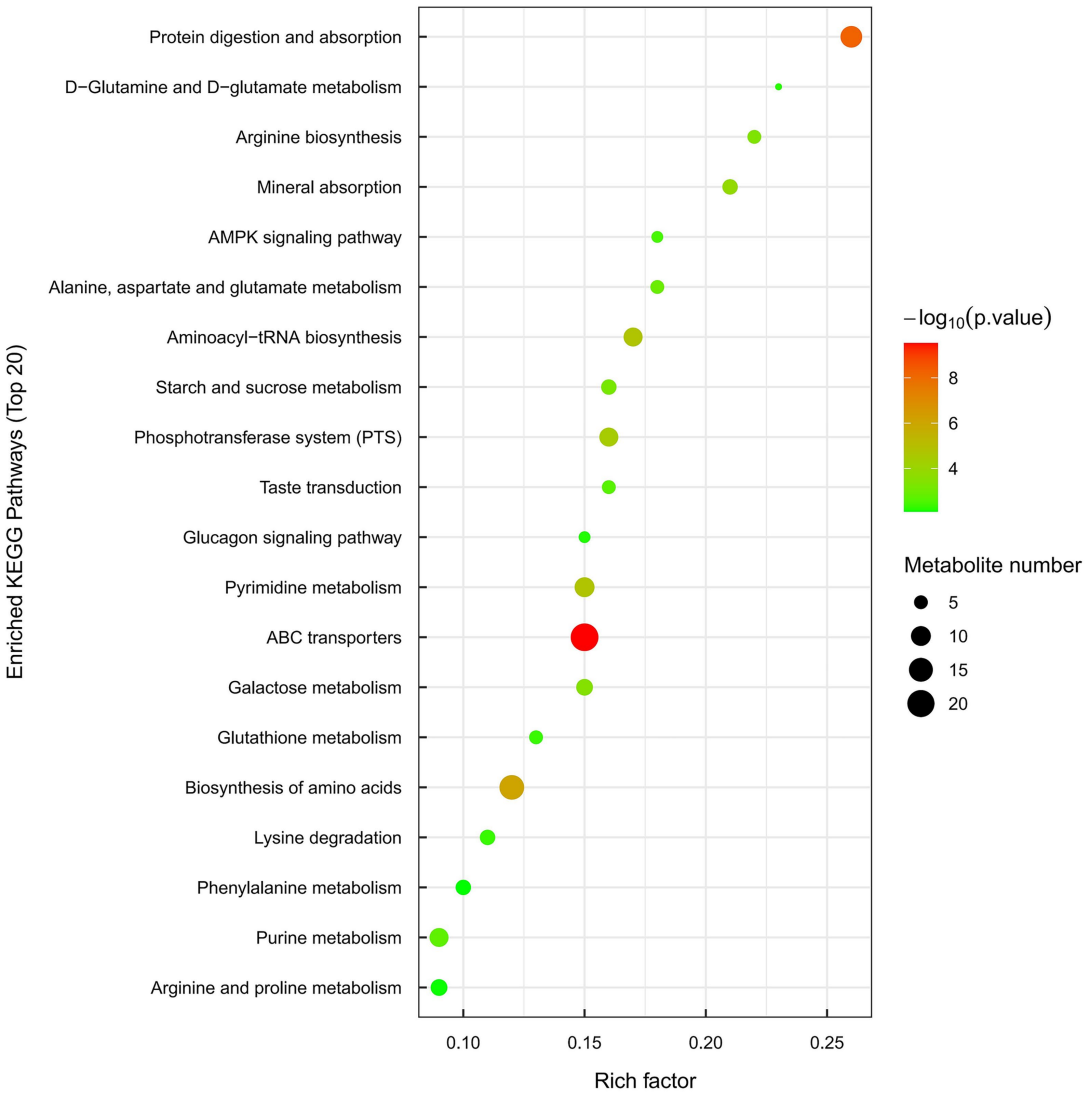
KEGG enrichment pathway (bubble chart). Each bubble in the figure means a metabolic pathway (the top 20 pathways selected with the highest significance based on *p-*value). The horizontal axis where the bubble is located and the size of the bubble means the extent of the impact factor of the pathway in topological analysis. The larger the size, the greater the impact factor. The color of the bubble indicates the *p*-value of enrichment analysis (using -log_10_^*p*-value^) along the vertical axis. The brighter the color, the smaller the *p*-value, and the more remarkable the enrichment level.

The results of the metabolic pathway enrichment analysis are summarized in the bubble chart shown in Figure 9. The analysis identified significant enrichment in multiple pathways, including: ABC transporters, protein digestion and absorption, amino acid biosynthesis, aminoacyl-tRNA biosynthesis, the phosphotransferase system (PTS), pyrimidine metabolism, purine metabolism, arginine biosynthesis, mineral absorption, starch and sucrose metabolism, alanine, aspartate and glutamate metabolism, arginine and proline metabolism, phenylalanine metabolism, and lysine degradation. Among these, the ABC transporters pathway demonstrated the highest enrichment significance (*p* < 0.001).

**Figure 10:**
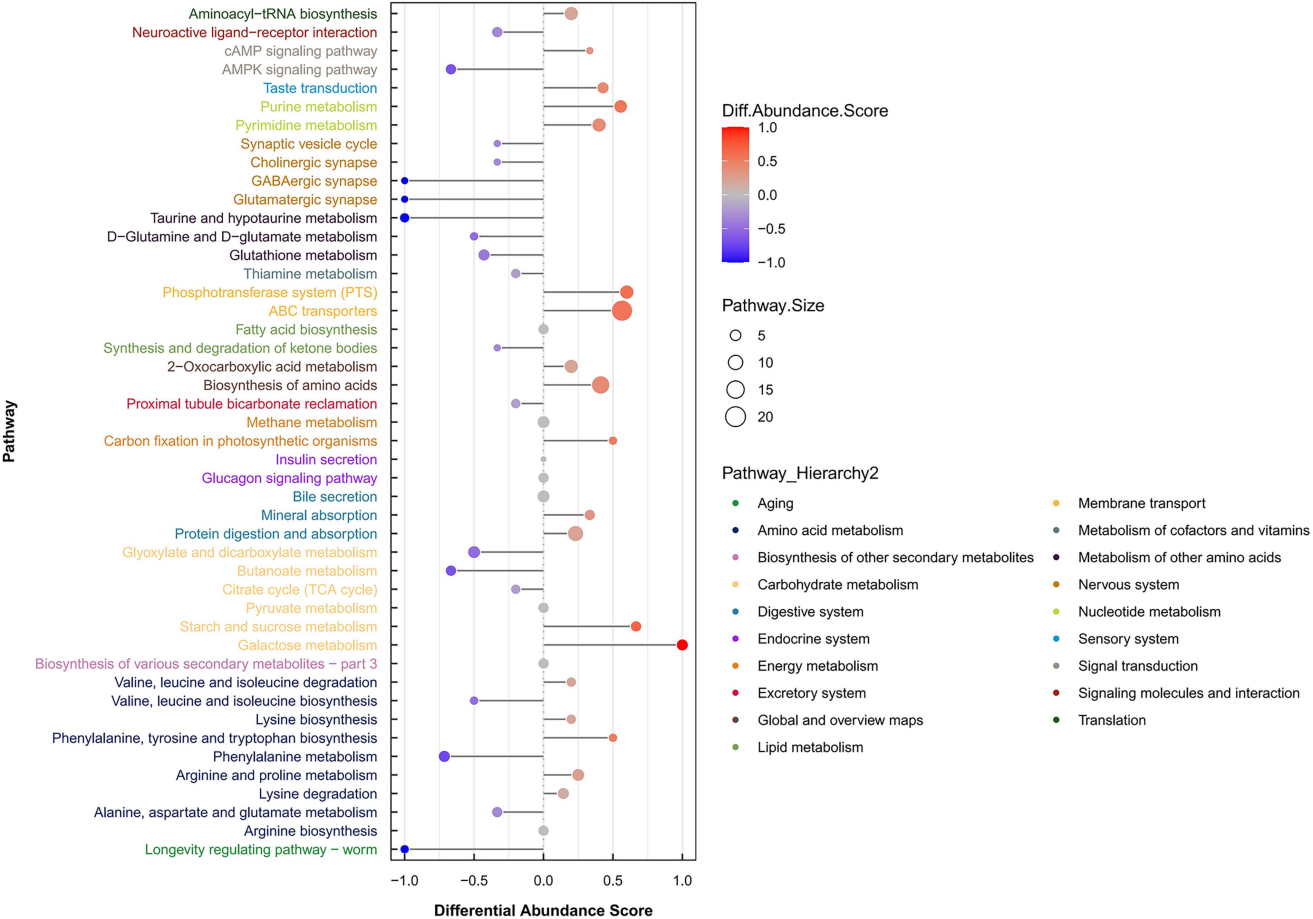
Differential abundance scores of all enriched metabolic pathways (classified according to pathway-hierarchy). The Y coordinate axis in the figure indicates the names of metabolic pathways, and the X coordinate axis coordinate indicates the differential abundance score (DA score). The DA score represents the overall change of all metabolites in pathway. A DA score of 1 means an upregulation of identified metabolites in this pathway, while a DA score of −1 means a downregulation of identified metabolites in this pathway. The length of the line segment indicates the absolute value of the DA score, and the size of the dots at the endpoints of the line segment indicates the number of metabolites in the pathway. The larger the dots, the more the number of metabolites. The length of line segments and the color of dots are proportional to the DA score value. The brighter the red color, the more likely the pathway is to be upregulated, and the bluer the color, the more likely the pathway is to be downregulated.

Metabolic pathway alterations were assessed using the Differential Abundance Score (DAS) method, which captures the overall directional change and average abundance shift of all metabolites within a given pathway. The DAS values for all enriched pathways are presented in Figure 10. The analysis revealed upregulation in several key pathways:

1. Amino acid metabolism: lysine degradation; arginine and proline metabolism; phenylalanine,tyrosine, and tryptophan biosynthesis.
2. Carbohydrate metabolism: galactose metabolism, starch and sucrose metabolism.
3. Global and Overview maps: amino acid biosynthesis and 2-oxocarboxylic acid metabolism.
4. Membrane transport: ABC transporters and the phosphotransferase system (PTS).
5. Nucleotide metabolism: pyrimidine metabolism and purine metabolism.
6. Signal transduction: cAMP signaling pathway.
7. Translation: aminoacyl-tRNA biosynthesis.

### The antagonistic effects of crude lipopeptide extracts and volatile compounds on *Fusarium graminearum*

Comparative analysis showed that the experimental group—treated with a crude lipopeptide extract from strain URR2—significantly suppressed the hyphal expansion of *F. graminearum* compared to the control during different phase of treatment. As time goes on, the inhibition of crude lipopeptides on the growth of *F. graminearum* hyphae becomes more significant. The maximum inhibition rate reached 63.86% after 96 h of antagonistic interaction (Figure 4B). Additionally, volatile compounds released by strain URR2 in liquid culture also inhibited the hyphal growth of *F. graminearum*. A statistically significant difference (*p* < 0.01) was observed relative to the control, with a peak hyphal inhibition rate of 30.38% after 48 h of exposure (Figure 4C).

### Microscopic observation of crude lipopeptide extract on the mycelial growth of *Fusarium graminearum*

**Figure 11:**
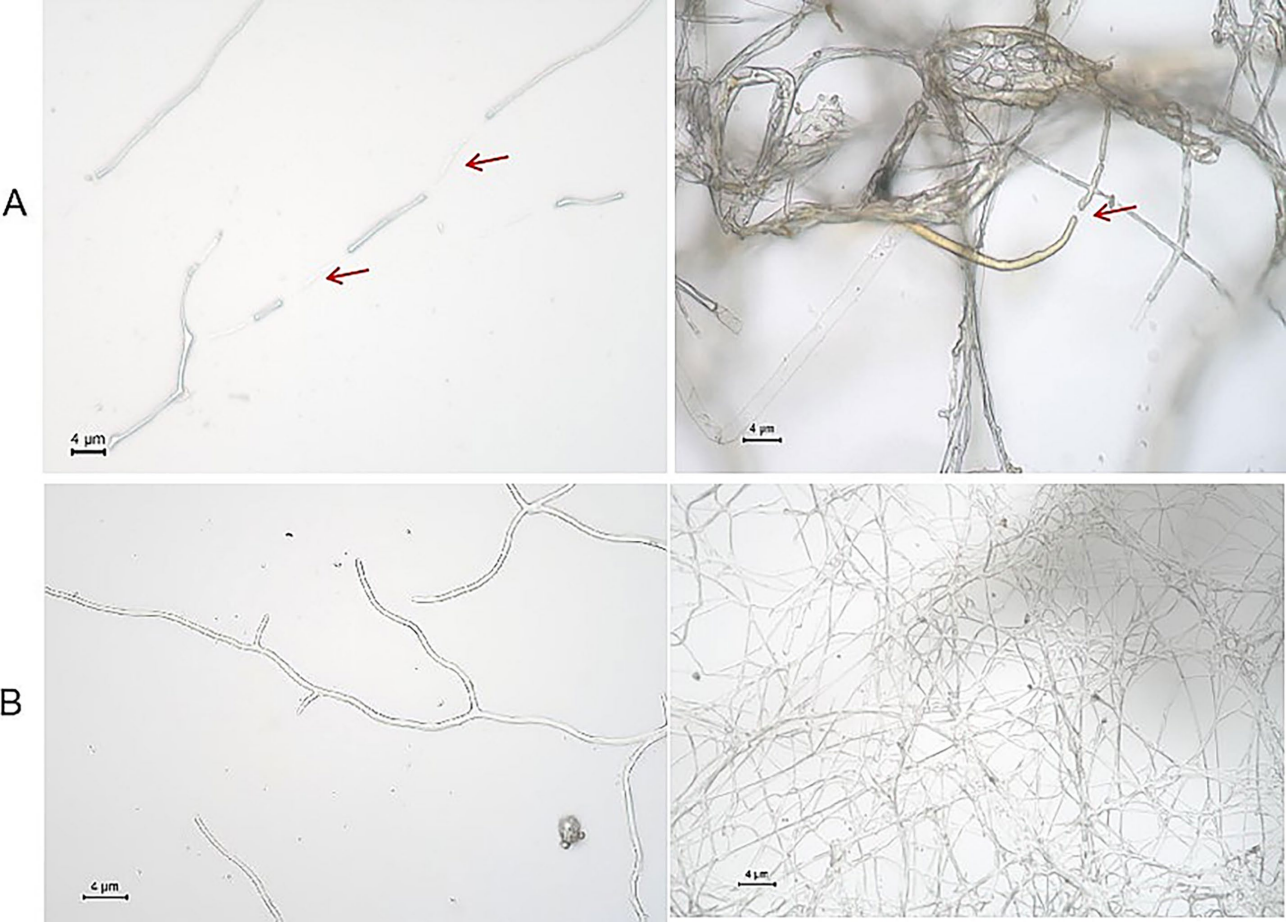
Morphological observation of *F. graminearum* PH-1 hyphae under different treatments. **(A)** Abnormal *F. graminearum* PH-1 hyphae (treatment group); **(B)** Normal *F. graminearum* PH-1 hyphae (control group). The red arrow indicates the rupture and enlargement of *F. graminearum* hyphae.

Lipopeptides extracted from strain URR2 were applied to the periphery of *F. graminearum* PH-1 colonies and co-cultured for 48 h. The treatment resulted in significant inhibition of fungal growth, characterized by reduced mycelial density and structural degradation. Fluorescence microscopy revealed marked morphological abnormalities in the treated hyphae including swollen hyphal junctions, fragmentation of hyphal strands, yellowing and necrosis of hyphal tissue, and complete cessation of normal growth. In contrast, control group hyphae maintained intact morphology and typical growth patterns (Figure 11). These findings conclusively demonstrate the potent antifungal activity of strainURR2-derived lipopeptides against *F. graminearum* PH-1.

### Identification of antimicrobial lipopeptides

**Figure 12:**
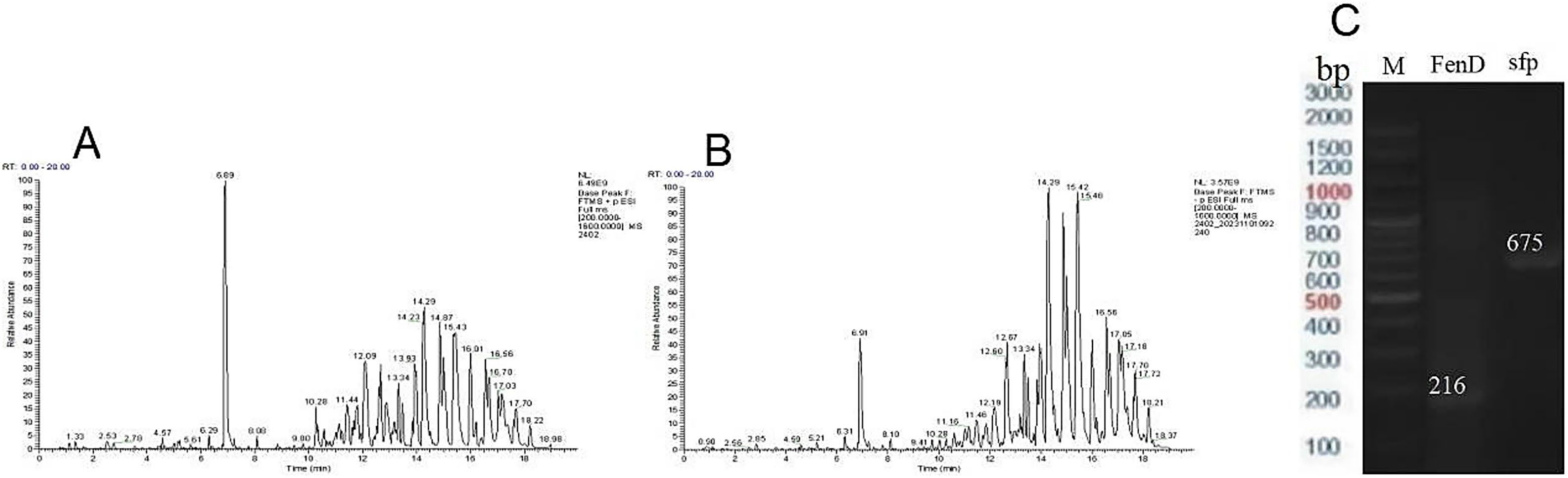
Peak chromatogram of crude extract of lipopeptide in positive ion mode **(A)** and in negative ion mode **(B)**, and detection of the lipopeptide biosynthesis genes in URR2 **(C)**.

Crude lipopeptide extracts from strain URR2 were subjected to mass spectrometry analysis. Spectral profiles were cross-referenced with published literature to identify the presence of surfactin and fengycin lipopeptide families (Figures 12A,B). Quantification revealed 10 distinct surfactin isoforms and 7 fengycin variants, with detailed molecular characteristics summarized in Table 3. Furthermore, to confirm the presence of genes involved in the biosynthesis of the antifungal lipopeptides surfactin (sfp) and fengycin (FenD), PCR amplification was performed on the URR2 genomic DNA with the selected primer pairs, which yielded the expected specific bands (Figure 12C).

**Table 3:** Chemical composition of lipopeptide substances from strain URR2.

Family	m/z	Z	Assignment	Retention time (min)	Peak area	Peak area percentage (%)
Surfactin	994.6458	1	C13 Surfactin [Val7] [M + H]+	16.21	6.64E+08	0.67
	1008.6609	1	C13 Surfactin [Leu7/Ile7] [M + H]+	16.01	2.00E+10	20.17
	1022.6762	1	C14 Surfactin [Leu7/Ile7] [M + H]+	16.56	1.32E+10	13.31
	1036.6933	1	C15 Surfactin [Leu7/Ile7] [M + H]+	17.03	7.52E+09	7.58
	1050.7009	1	C16 Surfactin [Leu7/Ile7] [M + H]+	17.62	3.26E+09	3.29
	1064.7205	1	C17 Surfactin [Leu7/Ile7] [M + H]+	18.22	3.87E+09	3.90
	1008.6609	1	C13 Pumilacidin [Val7][M + H]+	16.78	7.99E+08	0.81
	1022.6762	1	C14 Pumilacidin [Val7][M + H]+	17.29	2.84E+09	2.86
	1036.6933	1	C15 Pumilacidin [Val7][M + H]+	17.69	7.23E+09	7.29
	1050.7009	1	C16 Pumilacidin [Val7] [M + H]+	18.57	1.29E+08	0.13
Fengcin	725.3979	2	C15 Fengcin A[M + H]+	10.91	7.12E+08	0.72
	732.4062	2	C16 Fengcin A[M + H]+	11.44	1.11E+09	1.12
	739.4140	2	C17 Fengcin A [M + H]+	11.80	9.37E+09	9.45
	732.4062	2	C14 Fengcin B[M + H]+	11.36	8.12E+08	0.82
	739.4140	2	C15 Fengcin B[M + H]+	11.24	2.03E+09	2.05
	746.4223	2	C16 Fengcin B[M + H]+	11.68	2.60E+09	2.62
	753.4310	2	C17 Fengcin B[M + H]+	12.09	2.30E+10	23.20

### Identification of volatile organic compounds

Volatile organic compounds (VOCs) produced by strain URR2 were analyzed using headspace-gas chromatography-ion mobility spectrometry (HS-GC-IMS). Ten major VOCs were identified, including acetone, ethanol, trichloromethane, pyruvic acid, propadiene, hydrogen azide, 3-chlorobenzoyl acetonitrile, ammonium chloride, thioacetic acid, and 2-methyl-4- (2-methylpropyl) phenylacetic acid. The total proportion of acetone and ethanol content is 94.09%, with acetone having the highest proportion at 56.69%, followed by ethanol at 37.4% (Table 4).

**Table 4:** Top VOCs determination by HS-GC-IMS.

Classification	Molecular formula	CAS	Characteristic ion	Peak time	Peak area	Proportion (%)
Acetone	C_3_H_6_O	67–64-1	43	11.038	417162.35	56.69
Ethanol	C_2_H_6_O	64–17-5	45	10.23	275179.22	37.40
Bromochloromethane	CH_2_BrCl	67–66-3	83	15.598	10831.62	1.47
Pyruvic acid	C_3_H_4_O_3_	127–17-3	43	14.634	5508.56	0.75
Propadiene	C_3_H_4_	463–49-0	40	16.033	2883.18	0.39
Hydrogen azide	HN3	7,782-79-8	43	16.147	100.01	0.01
3-chlorobenzoyl Acetonitrile	CH_5_NO	67–62-9	47	16.582	99.26	0.01
Ammonium chloride	ClH_4_N	12,125–02-9	36	17.121	139.20	0.02
Thioacetic acid	C_2_H_4_OS	507–09-5	43	18.085	84.03	0.01
2-methyl-4- (2-methylpropyl) phenylacetic acid	C_13_H_18_O_2_	15,687–27-1	206	23.754	145.26	0.02

## Discussion

Endophytic bacteria represent a vast reservoir of diverse antimicrobial compounds (Ali et al., 2024). In recent years, endophytic bacteria have garnered increasing interest among researchers due to their plant growth-promoting properties and antagonistic activities (Patel et al., 2024). The deployment of endophytic bacteria as biocontrol agents presents an economically and environmentally viable alternative for phytopathogen management, reducing reliance on chemical pesticides. *Bacillus* spp. is a type of Gram-positive bacteria inhabiting a large number of different habitats with many characteristics, such as promoting plant growth through nitrogen fixation and secretion of auxin, reducing the amount of heavy metal in the environment, and increasing plant resistance against pathogens (Yousuf et al., 2017; Wróbel et al., 2023; Jeżewska-Frąckowiak et al., 2018). Bacteria of the genus *Bacillus* are promising biocontrol agents against phytopathogenic fungi, owing to their ability to synthesize a wide array of antimicrobial substances, which play important roles in stimulating defense mechanisms of plants (Mohammed et al., 2014; Fira et al., 2018; Gu et al., 2017; Yu et al., 2021; An et al., 2024). In this study, two endophytic bacterial strains, URR1 and URR2, were isolated from upland rice roots and identified as *Pseudomonas* sp. and *Bacillus subtilis*, respectively. Both strains demonstrated strong *in vitro* antagonistic activity against *F. graminearum*. Elucidating the antagonistic mechanisms of *B. subtilis* against *F. graminearum* is essential for its effective use as a biocontrol agent. Previous studies have reported that various *B. subtilis* strains exhibit biocontrol potential against *F. graminearum*. This antagonistic activity is primarily mediated through the production of diverse secondary metabolites and broad-spectrum antimicrobial compounds, such as lipopeptides and chitinase (Zhao et al., 2014). Bacterial endophytes belonging to *Bacillus* spp. were isolated from maize seeds and demonstrated antifungal activity against *Fusarium moniliforme*, exhibiting an inhibition zone at a concentration of 500 μg per disk. Antifungal compounds, including iturin A, fengycin, and bacillomycin, were identified in the isolates using MALDI-TOF mass spectrometry (Gond et al., 2015). Lipopeptides are amphiphilic, membrane-active peptide antibiotic with high activity against phytopathogens in small doses, causing damage and deformation of the filamentous membrane and cell wall of *F. graminearum* (Harish et al., 2023; Hanif et al., 2019). *In vitro* antagonistic assays confirmed the inhibitory efficacy of lipopeptides from *B. subtilis* strain Y17B against *Alternaria alternata* (Ahmad et al., 2023). Our findings demonstrate that *B. subtilis* URR2 primarily suppresses *F. graminearum* through the secretion of lipopeptides. Even at low concentrations (v/v 1%), the lipopeptides exhibited significant inhibitory effects on the hyphal growth of *F. graminearum*.

*Bacillus subtilis* likely compromises fungal cell membrane integrity through the secretion of these lipopeptides, resulting in the leakage of cellular contents (Wang et al., 2024). Consistent with this, our antagonistic assays demonstrated visible hyphal breakage and growth inhibition in *F. graminearum* following lipopeptide treatment. The lipopeptide profile of strain URR2 was analyzed in this study, revealing surfactins and fengycins as its major products. The sfp and fenD genes, which are responsible for the biosynthesis of the lipopeptides surfactin and fengycin, respectively, were successfully validated in the present work. Fengycins, which are cyclic lipodecapeptides, have been recognized as key antifungal lipopeptides in other *B. subtilis* strains effective against *F. graminearum* (Ramarathnam et al., 2007; Romanenko et al., 2008; Kim et al., 2017). Surfactins are known to synergistically enhance the antifungal activity of other lipopeptides (Hiraoka et al., 1992). Therefore, the co-production of both fengycins and surfactins likely contributes significantly to the efficacy of strain URR2 in controlling *F. graminearum*. Furthermore, Antagonistic experiments conducted in this study revealed that volatile organic compounds exhibit significant inhibitory effects. In previous studies, volatile organic compounds (VOCs), such as ketones, aromatic compounds, furan, pyrazine, alcohols, and ester, emitted by *Bacillus* species have been shown to exhibit antifungal activity against *F. oxysporum* and *F. graminearum* (Al-Mutar et al., 2023; Zhang et al., 2020; Yuan et al., 2012). Moreover, several volatiles produced by *Bacillus* strains, including acetophenone, 2-nonanone, m-tolunitrile, 2-ethylhexanol, 2-heptanone, benzylacetone, 6-methyl-2-heptanone, benzothiazole, 5-methyl-2-hexanone, dimethyl disulfide, dimethyl trisulfide, 1-undecene, benzaldehyde, cyclohexanol, and 2-ethyl-1-hexanol, have been demonstrated to inhibit fungal growth (Zhang et al., 2020; Kai et al., 2009).

Headspace with Gas Chromatography–Mass Spectrometry (HS-GC–MS) analysis is well applied in determination of volatile organic compounds (VOCs) (Castell et al., 2023). A range of volatile organic compounds such as acetone, ethanol, bromochloromethane, pyruvic acid, and propadiene were detected using HS-GC–MS in the present work. Some reports showed that VOCs poduced by *B. megaterium* significantly inhibited the mycelial growth of *F. graminearum* PH-1 (Keyfoğlu et al., 2023). A total of 21 high-concentration volatile organic compounds (VOCs) emitted by *B. thuringiensis* G-5 were identified using headspace gas chromatography–ion mobility spectrometry (HS-GC-IMS). Among these, 3-hepten-2-one was determined to be the primary antifungal component (Mo et al., 2025). Many of potential antifungal VOCs, comprising alcohols, ketones, pyrazines, esters, acids, phenols, amines, and hydrocarbons, were identified during the fermentation process. The composition and abundance of these VOCs varied across different fermentation time points (Gao et al., 2018). Our findings align with previous research, as acetone, ethanol, bromochloromethane, and pyruvic acid were identified as the predominant VOCs in this study, demonstrating strong antifungal activity. The strongest antifungal activity was recorded in the 48-h fermentation sample. The antifungal effects of these volatiles are likely mediated through mechanisms such as disruption of fungal cell membrane integrity, structural damage to fungal cells, or interference with key metabolic pathways.

Metabolomic analysis revealed that the differentially upregulated metabolites were primarily distributed in lipids and lipid-like molecules, benzenoids, organic acids and derivatives, organic oxygen compounds, organoheterocyclic compounds, organic nitrogen compounds, nucleosides, nucleotides and analogs, lignans and neolignans and related compounds, as well as phenylpropanoids and polyketides. Among them, organic acids and lipids were represented in a high proportion. This high abundance may be a key factor contributing to the strong antagonistic activity of the URR2 strain against pathogenic fungi. It is well-documented that organic acids demonstrate strong anti-fungal activity (Barbero-López et al., 2020). Fatty acids and amino acids serve as essential precursors for lipopeptide biosynthesis (Wang et al., 2024). Under specific conditions, *B. subtilis* URR2 may enhance lipopeptide production by upregulating key metabolic pathways involved in fatty acid ester synthesis and amino acid metabolism. Polyketides, including bacillaene, difficidin, macrolactin, and amicoumacin, represent another major group of antimicrobial metabolites produced by *Bacillus* species (Wang et al., 2024). Strain URR2 may produce more polyketides through upregulating phenylpropanoids and polyketides metabolism to inhibit *F. graminearum*. In addition, enrichment analysis of metabolic pathways indicated upregulation in membrane transport systems, specifically ABC transporters and the phosphotransferase system (PTS). ABC transporters may play a key role in bacterial adaptation to biotic stress. Endophytic bacteria upregulate the expression of ABC transporters to enhance carbon source uptake and thereby support their own survival. Additionally, these transporters can facilitate the secretion of antimicrobial peptides that antagonize fungal pathogens. For example, in *Bacillus* species, ABC transporters mediate the export of secondary metabolites such as antibacterial substances (Ohki et al., 2003).

In summary, the antagonistic mechanism of *B. subtilis* against *F. graminearum* appears to be multifaceted. Beyond the well-documented roles of lipopeptides and volatile organic compounds, further investigation is required to elucidate the contributions of certain polyketides, antimicrobial proteins, antibiotics, and other metabolites.

## Conclusion

In this study, endophytic bactreial strain URR1 and URR2 were identified as effective biocontrol strains against Fusarium head blight (FHB) in upland rice. The endophytic bacterium URR2 was found to produce diverse antifungal compounds, including lipopeptides and volatile organic compounds (VOCs), which exhibited activity against *Fusarium graminearum*. The antagonistic mechanism appears to involve the upregulation of certain metabolites and the participation of key metabolic pathways such as ABC transporters and the phosphotransferase system (PTS). These results indicate that URR2 holds strong potential as a novel biocontrol agent for the management of FHB in rice. Further research is needed to optimize fermentation conditions for enhanced lipopeptide production, and extensive field trials will be essential to assess the efficacy and practicality of URR2-based microbial pesticides in controlling rice FHB.

## Data Availability

The datasets presented in this study can be found in online repositories. The names of the repository/repositories and accession number(s) can be found in the article/Supplementary material.

## Footnotes

1
http://blast.ncbi.nlm.nih.gov
