# Supplementary material for: Screening, isolation and characterization of endophytic bacteria from upland Rice for antagonism against Fusarium graminearum
Source: Front Microbiol. 2025 Dec 17;16:1717984. doi: 10.3389/fmicb.2025.1717984 (PMC12753520; doi:10.3389/fmicb.2025.1717984)
Supplement: Supplementary file 1 [file Data_Sheet_1.PDF]

Supplemental table. The details of medium formulations

Medium	Formulation
LB medium	10.00 g of tryptone, 5.00 g of yeast extract, 10.00 g of sodium chloride, 1000 mL of distilled water, pH 7.0 ~ 7.4
PDA medium	200g of peeled potatoes, 20g of glucose, 20g of agar, 1000mL of distilled water, natural pH
Combined nitrogen-fixing culture medium	0.4 g of potassium dihydrogen phosphate, 5.0 g of D-sodium gluconate, 0.1 g of dipotassium hydrogen phosphate, 0.2 g of magnesium sulfate, 0.1 g of sodium chloride, 0.02 of gcalcium chloride, 0.01 g of ferric chloride, 0.8 g of yeast extract, 0.002 g of sodium molybdate, 15 g of agar, 1000mL of distilled water, pH 6.8 ~ 7.1
NA medium	5g of peptone, 3g of beef extract, 5g of sodium chloride, 15 ~ 20g of agar, 1000mL of distilled water, pH 7.2 ~ 7.4
Solid soybean culture medium	20 g of soybeans were ground using a grinder, boiled for 30 minutes, and filtered through four layers of gauze. To the filtrate, the following components were added: 10 g of glucose, 5 g of soluble starch, 2 g of peptone, 2 g of yeast extract, 2 g of NaCl, 1 g of $CaCO_3$, 0.5 g of $MgSO_4 \cdot 7H_2O$, 0.5 g of KH_2PO_4, and 15 g of agar powder. The volume was adjusted to 1000 mL with distilled water, natural pH
MS medium	19.0g of KNO_3, 1.5g of NH_4NO_3,1.25g of K_2HPO_4, 0.5g of $MgSO_4 \cdot 7H_2O$, 0.25g of $CaCl_2 \cdot 2H_2O$, 0.83mg of KI, 6.2mg of H_3BO_3, 16.9mg of $MnSO_4 \cdot 4H_2O$, 8.6mg of $ZnSO_4 \cdot 7H_2O$, 0.25mg of $Na_2MoO_4 \cdot 2H_2O$, 0.025mg of $CuSO_4 \cdot 5H_2O$, 30.0g of Sucrose, agar 7.0g, distilled water 1000mL
